# Covalent tethering of photo-responsive superficial layers on hydrogel surfaces for photo-controlled release†
†Electronic supplementary information (ESI) available. See DOI: 10.1039/c6sc04634g



**DOI:** 10.1039/c6sc04634g

**Published:** 2016-11-16

**Authors:** Lie Chen, Xi Yao, Zhandong Gu, Kaikai Zheng, Chuangqi Zhao, Wenwei Lei, Qinfeng Rong, Ling Lin, Jiaobing Wang, Lei Jiang, Mingjie Liu

**Affiliations:** a Key Laboratory of Bio-Inspired Smart Interfacial Science and Technology of Ministry of Education , School of Chemistry and Environment , Beihang University , Beijing , 100191 , P. R. China . Email: liumj@buaa.edu.cn; b School of Engineering and Applied Sciences , Kavli Institute for Nanobio Science and Technology , Harvard University , Cambridge , 02138 MA , USA; c State Key Laboratory of Polymer Physics and Chemistry , Institute of Chemistry , Chinese Academy of Sciences , Beijing , 100190 , P. R. China; d School of Chemical and Chemistry Engineering , Sun Yat-sen University , Guangzhou , 510275 , P. R. China; e Key Laboratory of Bio-Inspired Materials and Interfacial Science , Technical Institute of Physics and Chemistry , Chinese Academy of Sciences , Beijing 100190 , P. R. China; f International Research Institute for Multidisciplinary Science , Beihang University , Beijing , 100191 , P. R. China; g Engineering Research Center of Marine Biological Resource Comprehensive Utilization , SOA , The Third Institute of Oceanography of the State Oceanic Administration , Xiamen 361005 , China

## Abstract

The diffusion and transport of substances between a hydrogel and its environment have received tremendous research interest, due to the wide range of applications of hydrogel materials in fields related to drug carriers and drug delivery vehicles. To date, much research has been done to tailor the diffusion and transport of substances through hydrogels, where most efforts were focused on tuning the 3D network properties of the hydrogel including loop size, hydrophobicity of building blocks and the stimuli-responsive properties of backbones. These conventional strategies, however, usually suffer from complicated fabrication procedures and result in a homogeneous increase in hydrophobicity of the hydrogel network, leading to low efficiency control over the diffusion of substances through the hydrogel. Herein, a facile strategy that can functionalize the surfaces of hydrogels, while keeping the interior network unchanged, was reported, and is realized by quaternization reaction confined to the hydrogel/oil interface. Owing to the introduction of the photo-responsive molecule IBSP as a modifier, the surface wettability of the resulting hydrogel can be controlled by light both in air and underwater environments. Consequently, the diffusion rate of a substance through this modified hydrogel can be regulated by light, which brings convenience to the controlled release of hydrogels and other hydrogel-related fields.

## Abstract 2

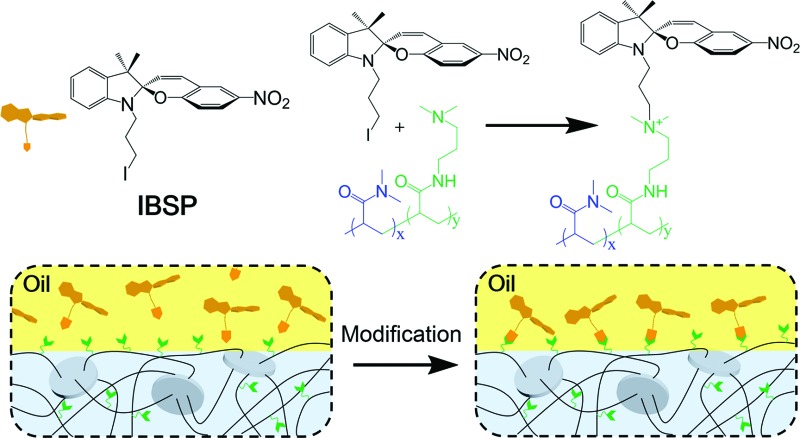
A facile strategy that can functionalize the surfaces of hydrogels, while keeping the interior network unchanged, was reported.

## Introduction

Hydrogels, composed of 3D crosslinked hydrophilic polymers and a large amount of water, have been widely used in areas related to tissue engineering, cellular immobilization, drug carriers and drug delivery vehicles due to their unique physical and chemical properties. In these applications, the substances’ diffusion and exchange between the hydrogel and its environment has vital significance. For decades, a lot of research has been done in order to realize the controlled release of hydrogels through tuning the 3D network properties such as loop size and hydrophobicity of building blocks.^1,2^ For example, hydrogels that release drugs can be continuously regulated by controlling the ratio of hydrophilic hydrogen bonds and hydrophobic π–π stacking.^3^ A physically crosslinked hydrogel, composed of hydrophilic components and hydrophobic components, has proven to be effective in controlling the desorption rate of a drug substance from its matrix.^4^ These strategies focusing on tuning the inner network properties of hydrogels, however, usually result in a homogeneous increase in hydrophobicity, leading to low efficiency control over the substance diffusion through hydrogels. In contrast, in biological processes the hydrophobic interfaces, such as cell membranes and certain nucleoporin hydrogels, play a key role in controlling diffusion.^5^ Nevertheless, using hydrophobic surfaces on hydrogels to solve this problem has often been underestimated.

To date, a large variety of stimuli-responsive hydrogel systems that can respond to external stimuli, such as light illumination,^6^ temperature,^7^ pH,^8^ ionic strength,^9^ electricity,^10^ magnetic fields,^11^ and certain chemicals, were developed for the controlled release of cargoes embedded in hydrogels.^12^ Light and temperature are contact free and environmentally friendly stimuli, which make them the most commonly used to trigger substance release from hydrogels. Conventionally, the most straightforward method to obtain a stimuli-responsive hydrogel is the chemical modification of a hydrogel with stimuli-responsive moieties in its building blocks or use of a stimuli-responsive polymer to construct its network.^13^ To realize the controlled release of small molecules or macromolecules such as proteins and other biomacromolecules based on this hydrogel, stimuli-responsive swelling/deswelling and sol–gel transition processes of the hydrogel were generally adopted.^14^ However, these strategies that are used to prepare stimuli-responsive hydrogels usually require complex procedures, and the sol–gel transition process of the hydrogel would inevitably lead to the burst release of cargoes incorporated in the hydrogel. Consequently, it remains a challenge to develop a more reasonable strategy that can overcome these shortcomings.

Herein, we report a facile strategy to functionalize the surface of hydrogels with photo-responsive wettability and photo-controlled release properties. A spiropyran derivative was covalently tethered on the hydrogel surface forming a superficial layer with a thickness of about 1.2 μm through confined quaternization reaction at the hydrogel/oil interface. The resulting hydrogel exhibits superhydrophobicity and photo-responsive properties on its surface, while the superhydrophilicity of the hydrogel inner networks was well preserved. Due to the photo-responsive ring opening and ring closing mechanisms of modifiers, the underwater oil wettability and underwater oil adhesion force upon this modified hydrogel can be reversibly switched using light illumination. Finally, this superhydrophobic photo-responsive superficial layer on the modified hydrogel has a significant influence on preventing the immediate diffusion of substances from the hydrogel to the aqueous environment, and the diffusion rate of substances from this modified hydrogel can be controlled using light. The simple method to prepare stimuli-responsive hydrogels reported in this work promotes immediate applications of hydrogels in areas related to substance diffusion, as well as potentially having applications in drug carriers or drug controlled release systems.

## Results and discussion

**Fig. 1:**
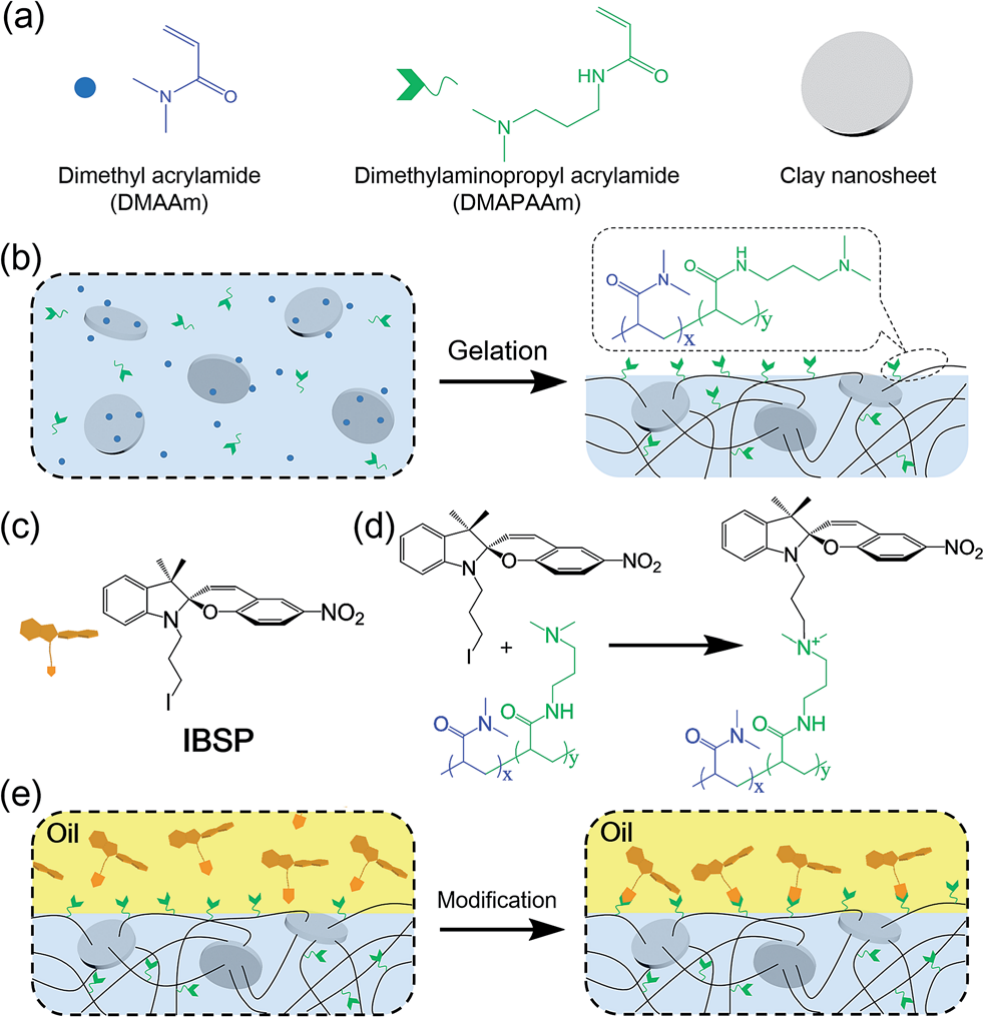
Schematic illustration of the preparation and modification of the hydrogel. (a) Common monomers (blue), anchor monomers (green) and clay nanosheets (crosslinker) were used to prepare the nanocomposite hydrogel. (b) All monomers and crosslinkers were homogeneously dispersed in water and, after the gelation process, the surface of the resulting hydrogel was covered with dimethylamino groups (active points). (c) Molecular formula of the photo-responsive molecule IBSP. (d) The modification process was realized through quaternization reactions. (e) Schematic illustration of the modification using IBSP as a modifier at the hydrogel/oil interface (with a limited thickness on the hydrogel).

Fig. 1a shows the general components used to prepare the hydrogel, where *N*,*N*-dimethylaminopropyl acrylamide (DMAPAAm) is utilized to provide tertiary amine groups (active grafting sites) on the surface of the hydrogel.^15^ The hydrogel is prepared by copolymerizing DMAPAAm with *N*,*N*-dimethylacrylamide (DMAAm), using clay nanosheets (LAPONITE® XLS) as a physical crosslinker.^16^ As shown in Fig. 1b, tertiary amine groups were generated on the surface and inside of the as-prepared hydrogel. In order to achieve photo-responsive properties of the hydrogel, IBSP molecules were utilized as modifiers, and their structure is shown in Fig. 1c.^17^ As for the modification of the hydrogel, the *n*-alkylation reaction is introduced in our work, which was extensively used to graft target groups on tertiary amines.^18^ The as-prepared hydrogel was immersed in an oil phase (dichloromethane) in which a certain concentration of modifiers was dissolved. Due to the high water content (80 wt%) of the hydrogel, oil cannot penetrate into the hydrogel network. As a result, the *n*-alkylation reaction (Fig. 1d) was confined to only take place at the surface of the hydrogel, as illustrated in Fig. 1e.^19,20^ It is worth noting that the oil used in this work (dichloromethane) is not perfectly immiscible with water (the solubility of dichloromethane in water is 13.2 g L^–1^ at 25 °C),^21^ which would give rise to the modifiers dissolved in oil penetrating into the hydrogel and forming a modified layer with limited thickness on the hydrogel surface. The thickness of this hydrophobic layer will be discussed later in this work (Fig. 3a and b).

**Fig. 2:**
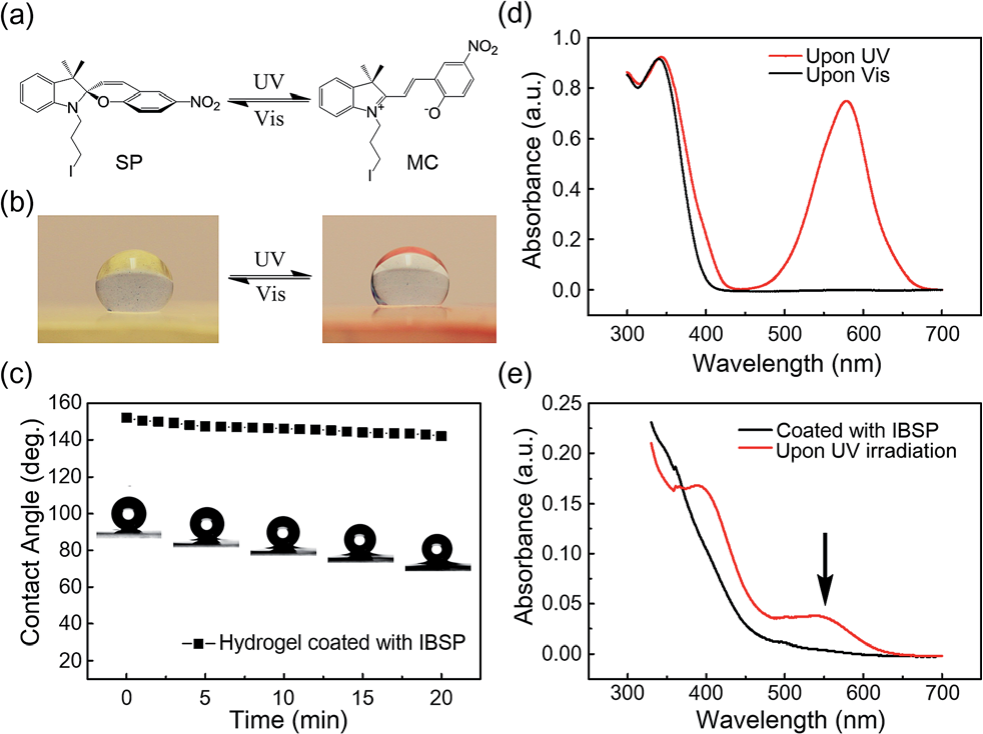
The characterization of wettability on the IBSP-modified hydrogel. (a) Photo-responsive properties of photochromic molecule IBSP. (b) Photographs of a water drop (10 μL) sitting on an IBSP-modified hydrogel (1 × 1 × 1 cm^3^) under visible (left, 470 nm, 30 mW cm^–2^, 5 min) and UV (right, 365 nm, 120 mW cm^–2^, 10 s) irradiation. Due to the photochromic properties of IBSP molecules, the color of the hydrogel surface is obviously changed (the contact angles were 141.1° (left) and 123.6° (right)). (c) A series of snapshots recording a droplet of water (2 μL) spreading on an IBSP-modified hydrogel. (d) UV-Vis spectra of IBSP dissolved in dichloromethane. (e) UV-Vis spectra of an IBSP-modified hydrogel slice (1 mm in thickness).

The photo-responsive molecule IBSP that is used in this work is basically of the same nature as spiropyran, which is a widely utilized photo-responsive molecule, due to the vast difference in physico-chemical properties of spiropyran (SP) and merocyanine (MC) isomers.^22^ The charge separation in MC that is formed upon ultraviolet (UV) irradiation gives rise to a large electric dipole moment compared with the SP isomer. As shown in Fig. 2a, the SP and MC isomers can be reversibly switched through illumination with UV and visible (Vis) light, respectively.

As shown in Fig. 2b, the surface of the hydrogel became superhydrophobic after modification,^23^ and when further illuminated with UV (365 nm, 120 mW cm^–2^, 10 s) irradiation the hydrophobicity of the hydrogel surface was reduced, which is due to the photo-induced isomerization of the non-polar SP form to the polar MC form. This process can be reversed by visible light (470 nm, 30 mW cm^–2^, 5 min) illumination.^24^ As shown in Fig. 2c, the water contact angle on the surface of the modified hydrogel was decreased by 10 degrees (from 152.1° to 142.1°) in twenty minutes. This indicated that the superhydrophobicity of the modified layer on the hydrogel is quite stable in air.

The success of interfacial modification of the hydrogel was confirmed using UV-Vis spectroscopy. Fig. 2d shows UV-Vis spectra of IBSP (dissolved in dichloromethane) before (black) and after (red) UV irradiation, and it was found that the SP isomer is optically transparent in the visible region whereas the MC isomer absorbs strongly at 500–650 nm. Fig. 2e shows UV-Vis spectra of the hydrogel (*ca.* 1 mm in thickness) coated with IBSP, and after UV irradiation it develops a broad peak at 500–600 nm, which is almost the same position as compared with Fig. 2d. This suggested that the IBSP was successfully coated on the hydrogel. In addition, the success of modification can also be identified by the color change of the modified hydrogel before (faint yellow) and after (pink) UV irradiation (Fig. 2b).

**Fig. 3:**
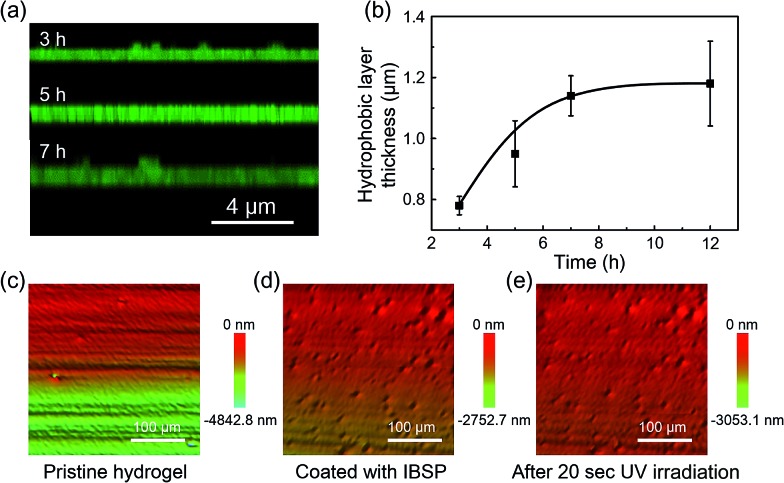
The characterization of the thickness of the modified layer and the surface topography of the hydrogel. (a) Laser scanning confocal microscopy observation shows that the thickness of the modified layer increased upon increasing the modification time. (b) The thickness of the modified layer as a function of the modification time. Upon extension of the modification time, the thickness of the modified layer first increased and then reached a plateau in about 7 hours. (c) Surface topography of the pristine hydrogel. (d) Surface topography of the IBSP-modified hydrogel, which became rougher compared with the pristine hydrogel. (e) Surface topography of the IBSP-modified hydrogel after *in situ* UV irradiation (365 nm, 120 mW cm^–2^, 10 s). No obvious change was observed compared with (d).

As mentioned above, the oil used in this work was not perfectly immiscible with water, which would give rise to the modifiers dissolved in oil being able to penetrate into the hydrogel and graft onto the polymer network, forming a hydrophobic layer with limited thickness on the hydrogel surface. The thickness of this hydrophobic layer was characterized using laser scanning confocal microscopy (LSCM). Owing to the fluorescence properties of IBSP (Fig. S1†), the fluorescence of IBSP molecules can be observed using LSCM when IBSP is illuminated by proper excitation light (488 nm). As shown in Fig. 3a, the thickness of the modified layer increased upon increasing the modification time. Furthermore, with an extension of the modification time, the thickness of the modified layer reached a plateau (1.2 μm) in about 7 hours (Fig. 3b).

Since the modification of the hydrogel was successful (the success of the modification was also confirmed using FTIR spectroscopy as shown in Fig. S2†), we supposed that the superhydrophobicity of the hydrogel was attributed to its surface composition and surface roughness. On one hand the surface energy was reduced due to the hydrogel surface being covered by hydrophobic components after modification.^25^ On the other hand, after modification hierarchical micro-/nanoscale surface roughness on the IBSP-modified hydrogel was generated. Fig. 3c and d show the 3D morphology of the hydrogel before and after modification, respectively. Before modification, only micro-scale grooves were observed, which are introduced by cutting. After modification, micro-/nanoscale surface roughness was generated and contributed to the formation of superhydrophobicity on the surface of the modified hydrogel.^26^ As for the formation of micro-/nanoscale surface roughness on the IBSP-modified hydrogel, it was supposed that the organic modified layer was swollen with oil after the modification. Once the hydrogel was exposed to an air environment, this layer would shrink and form nano-scale roughness due to oil evaporation.^27^ Further, upon illuminating the IBSP-modified hydrogel with UV light there was no obvious difference observed in the surface morphology, as shown in Fig. 3e compared with Fig. 3d. This may be due to the change in surface morphology on a molecular scale, which cannot be observed using 3D optical microscopy.

**Fig. 4:**
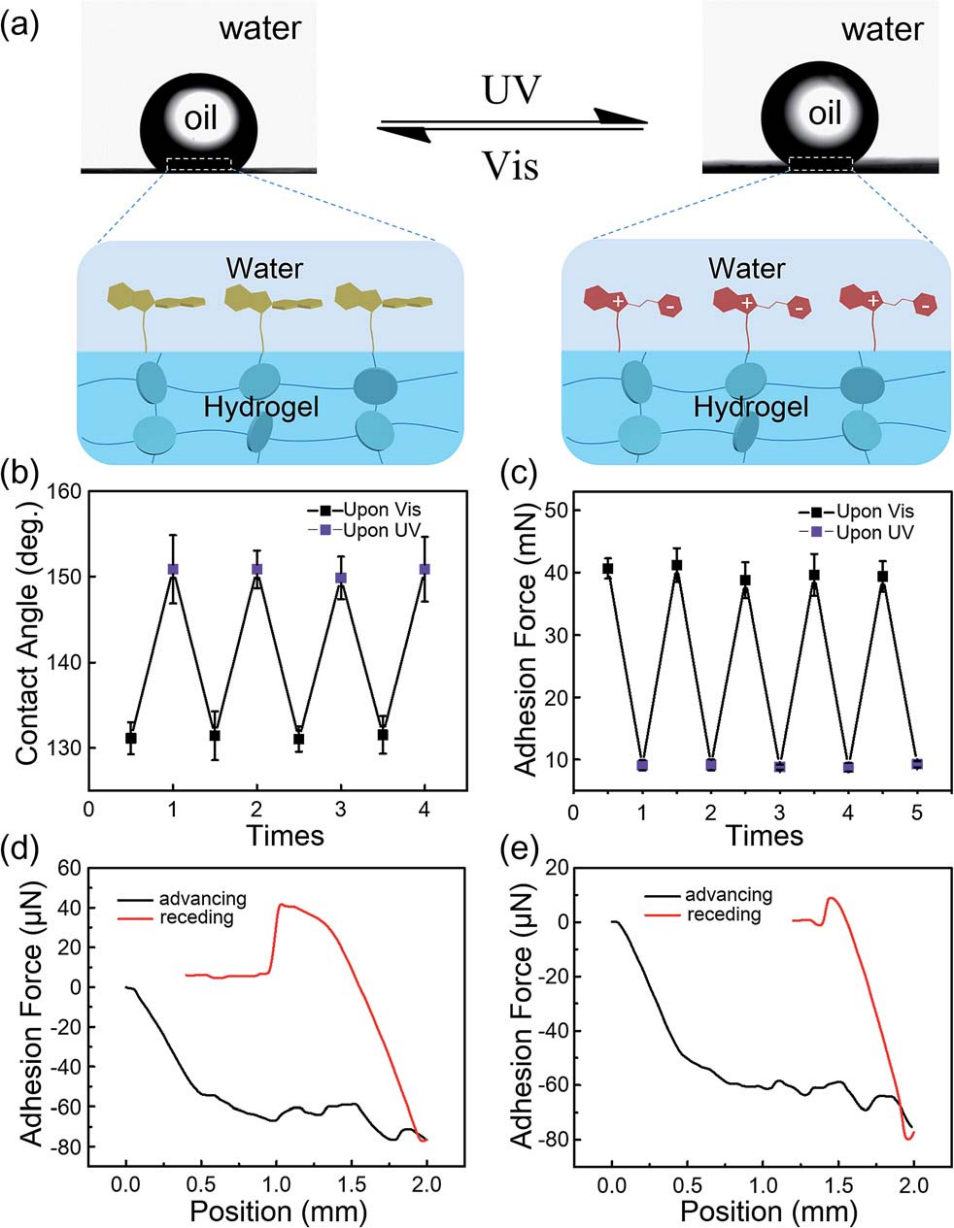
Underwater photo-responsive properties of the IBSP-modified hydrogel. (a) In an underwater environment, the oil (1,2-dichloroethane, 2 μL) contact angle (OCA) on the IBSP-modified hydrogel can be reversibly switched by UV (365 nm, 120 mW cm^–2^, 20 s) and Vis (470 nm, 30 mW cm^–2^, 10 min) illumination. (b) Photo-responsive reversible switch of the underwater OCA on the IBSP-modified hydrogel. (c) Photo-responsive reversible switch of the underwater oil (5 μL) adhesion force on the IBSP-modified hydrogel. Force–distance curves during the underwater oil adhesion force measurements (d) before and (e) after UV irradiation.

Generally, due to the dehydration of hydrogels in air, hydrogels have been designed and prepared for use in aqueous environments, such as in microfluidic devices,^28^ water/oil separation,^29^ contact lenses,^30^ drug release systems and marine antifouling coatings.^31^ Taking this into consideration, a few tests were carried out in underwater environments to estimate the stability of the modified layers on hydrogel surfaces. As shown in Fig. 4a, the underwater oil (1,2-dichloroethane, 2 μL) contact angle on the IBSP-modified hydrogel became larger upon UV irradiation, which indicates an increase of oleophobicity of the IBSP-modified hydrogel. The wettability change in this liquid/liquid/solid system is attributed to the existence of IBSP on the hydrogel surface. Upon UV irradiation, the non-polar SP isomer on the hydrogel surface changed to a strong polarity MC form, which can be easily wetted by water as illustrated in Fig. 4a. Accordingly, once the IBSP-modified hydrogel was illuminated by UV light in an underwater environment, the surface of the hydrogel was rapidly wetted by water, which prevented the surface from being further wetted by oil, and thus gave rise to a large underwater oil contact angle (OCA) on the hydrogel. Owing to the reversible photo-responsive properties of IBSP, this process can be reversed by visible light illumination. Fig. 4b shows the reversible switching of the underwater OCA on the IBSP-modified hydrogel upon UV (150.6 ± 4.3°) and Vis (131.3 ± 3.1°) light irradiation, respectively. Moreover, the underwater oil (1,2-dichloroethane, 5 μL) adhesion force upon the IBSP-modified hydrogel can also be reversibly switched, along with the change in wettability.^32^ In the experiment, the adhesive force was defined as the force required to take the oil drop away from the substrate. An oil droplet was brought into contact with the surface of the hydrogel then allowed to leave in an underwater environment, and the adhesion force between the oil and the surface was recorded using a high-sensitivity micromechanical balance system.^32^ Force changes were recorded using a balance system when the hydrogel surfaces were controlled to come into contact with and leave an oil drop. An optical microscope lens and a charge-coupled device (CCD) camera system were also used to record images during the measurements. As shown in Fig. 4c, upon Vis light illumination, the underwater oil adhesion force upon the IBSP-modified hydrogel was about 40 mN, while after UV irradiation this force decreased by about 30 mN. The corresponding force–distance curves of the underwater oil adhesion force measurement before and after UV irradiation are shown in Fig. 4d and e, respectively. As a supplement, a series of snapshots corresponding to the four stages of the dynamic underwater oil adhesion force measurements are shown in Fig. S3,† which also represents the change of underwater oil adhesion force before and after UV irradiation. It is noted that each photo-responsive reversible switch process (Fig. 4b and 3c) can be carried out at least four times, suggesting that the superficial modified layer on the hydrogel is quite stable in an underwater environment and might be further used to manipulate the substances’ diffusion through the surface of the hydrogel.

**Fig. 5:**
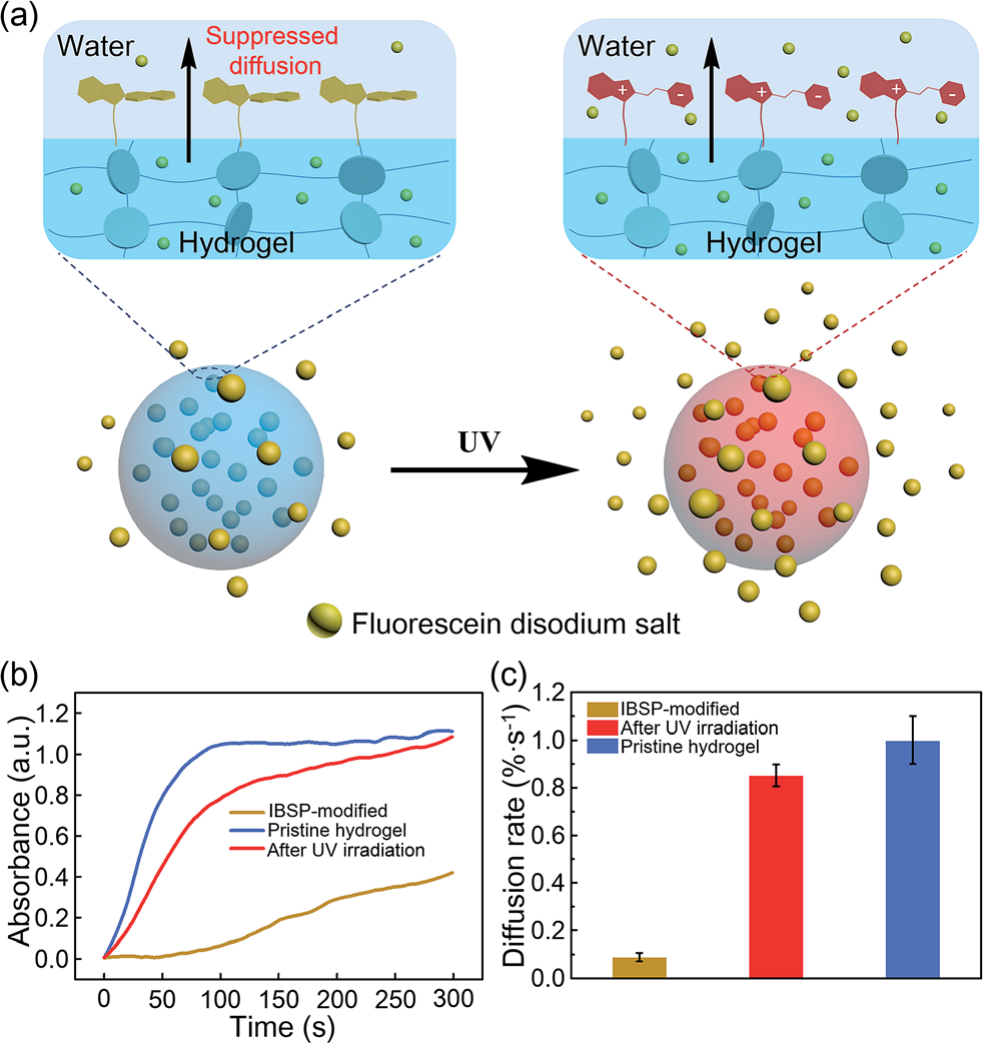
Photo-responsive controlled release of fluorescein. (a) Schematic of the photo-responsive release system. (b) *In situ* UV/Vis absorption at 490 nm (the characteristic peak of fluorescein disodium (Fig. S4†)) as a function of time, monitoring the fluorescein released to the water environment from the hydrogel. (c) The average diffusion rate of fluorescein was deduced from (b) during the first hundred seconds.

To show that this modified layer of the hydrogel can indeed act as a “smart” barrier during the substances’ diffusion between the hydrogel and aqueous environment, we designed a small molecule release system. The mechanism is illustrated in Fig. 5a. After modification, the surface of the resulting hydrogel was covered with a photo-responsive superhydrophobic IBSP layer, which was able to prevent the surface from being wetted by water and suppressed the cargoes inside the bulk hydrogel to be released into the aqueous environment at a certain time. Once the modified hydrogel was illuminated by UV light, the surface of the modified hydrogel became much more easily wetted by water due to isomerization of the non-polar SP form to the polar MC form. Consequently, the diffusion rate of substances through the surface of the hydrogel was accelerated.

At first, fluorescein was embedded into the hydrogel network, and the rate of fluorescein being released from the hydrogel to aqueous solution was monitored using UV-Vis spectroscopy. The diffusion amount of fluorescein from the hydrogel as a function of time is shown in Fig. 5b, and the pristine hydrogel did not exhibit suppression of the fluorescein release, which is due to the size of the fluorescein molecule being much smaller than the loop size of the hydrogel network, resulting in free diffusion of fluorescein from the hydrogel. In contrast, the diffusion of fluorescein from IBSP-modified hydrogels was significantly blocked. When further illuminated by UV light (365 nm, 120 mW cm^–2^, 20 s) the diffusion rate of fluorescein from the IBSP-modified hydrogel was accelerated. Fig. 5c shows the average diffusion rate of fluorescein released from the hydrogel, deduced from Fig. 5b (first 100 s). The average diffusion rate of fluorescein from the IBSP-modified hydrogel to water is ∼0.088% s^–1^, while after UV irradiation, the average diffusion rate is ∼0.853% s^–1^, which is about 10 times faster. These results show the excellent photo-responsive controlled release properties of the IBSP-modified hydrogel, which indicates that the IBSP-modified layer on the hydrogel can indeed act as a “smart” barrier during substances’ diffusion across the surface of the hydrogel. As for the long period release of this system, the IBSP-modified hydrogel also exhibited good photo-responsive controlled release properties (Fig. S5 and S6†). The time that is taken to release 60% of the total amount of fluorescein for the IBSP-modified hydrogel has been delayed by more than one hour compared with the pristine hydrogel, and this time for the IBSP-modified hydrogel can be further accelerated using UV irradiation at the initial stage of the release process.

## Conclusions

In summary, we present a strategy to functionalize the surface of a hydrogel to achieve photo-responsive wettability and photo-controlled release properties. IBSP was covalently tethered on the hydrogel surface, forming a superficial layer with a thickness of about 1.2 μm, through confined quaternization reaction at the hydrogel/oil interface. Results show that superhydrophobicity was achieved on the hydrogel surface, while the superhydrophilicity and biocompatibility of the inner hydrogel network was well preserved. Due to the introduction of photo-responsive IBSP as a modifier, the underwater oil wettability and oil adhesion force on the IBSP-modified hydrogel can be reversibly switched by alternately illuminating the IBSP-modified hydrogel with UV and visible light. Furthermore, this photo-responsive superhydrophobic superficial layer on the hydrogel surface can act as an intelligent valve to regulate substance diffusion between the hydrogel and its surrounding aqueous environment. Most importantly, unlike previous strategies to fabricate stimuli-responsive hydrogels, our method does not rely on the existence of a stimuli-responsive moiety in the building blocks of the hydrogel network, which makes it more advanced and applicable to a wide range of hydrogels. As a facile strategy to functionalize the surface of the hydrogel, this work may promote applications of hydrogels in areas related to biosensors, substance diffusion and transport through hydrogels, as well as potential applications in tissue engineering and controlled drug release systems.

## Experimental section

### Preparation of the hydrogel

In a typical procedure the anchor monomer, regular monomer and clay nanosheets (LAPONITE® XLS) were dissolved in water. Then the homogeneous precursor was initiated with potassium persulfate (K_2_S_2_O_8_), using *N*,*N*,*N*′,*N*′-tetramethylethylenediamine (TEMED) as a catalyst. The resulting hydrogel was transparent. Note that the tertiary amine on the anchor monomer could be unstable in an acidic environment, therefore alkaline ultrapure water (pH = 11) was used, which was obtained by adding NaOH to the water.

### Modification of the hydrogel

The modifiers (IBSP) were dissolved in dichloromethane (0.5 g mL^–1^). Then the as-prepared hydrogel was immersed in this organic solution and the *n*-alkylation reaction was conducted for various lengths of time (1–12 h), after which the hydrogel was rinsed with adequate CH_2_Cl_2_ to remove any non-chemically bonded residues. The resulting hydrogel was dried under nitrogen flow.

### Water and underwater oil contact angle measurement on the hydrogel surfaces

All contact angles were measured on the OCA20 Contact Angle Measuring System (Dataphysics). A 2 μL water/oil droplet was carefully deposited on the hydrogel surfaces using a hydrophobized syringe. At least five different spots on the same sample surface were used for contact angle measurements to obtain a mean value.

### Underwater oil adhesion force measurement

The adhesion force was measured using a high-sensitivity microelectromechanical balance system (Dataphysics DCAT 11, Germany). The force was measured in an aqueous environment. A dichloroethane (DEC) droplet of about 5 μL was suspended with a copper cap first, then a hydrogel sample (1 × 1 × 1 cm^3^) was placed on the balance table and the substrate with the hydrogel was moved upward at a constant speed of 0.005 mm s^–1^, until the underlying surface came into contact with the DEC droplet. Then the surface was moved down at the same speed. The adhesion forces were obtained from the force–distance curves. For each sample, the average adhesion force was obtained from five repeats.

### Preparation of the fluorescein-incorporated hydrogel

The fluorescein-incorporated hydrogel was obtained by dissolving a certain amount of fluorescein in the solution used for preparing the hydrogel (the fluorescein concentration in the resulting hydrogel was 0.0625 mg g^–1^). The polymerization was initiated with potassium persulfate (K_2_S_2_O_8_), using *N*,*N*,*N*′,*N*′-tetramethylethylenediamine (TEMED) as a catalyst (see ESI† for experimental details).

### UV spectrometric characterization of substance diffusion

The fluorescein-incorporated hydrogel was chopped into small cubes (3 × 3 × 3 mm^3^), which were treated through post-modulation. One hydrogel cube was immersed in water, contained in a colorimetric cuvette. The vertical distance between the hydrogel upper surface and the detection spot in the UV spectrometer remained constant.
