# Supplementary material for: Covalent tethering of photo-responsive superficial layers on hydrogel surfaces for photo-controlled release
Source: Chem Sci. 2016 Nov 16;8(3):2010–6. doi: 10.1039/c6sc04634g (PMC5398270; doi:10.1039/c6sc04634g)
Supplement: Supplementary file 1 [file SC-008-C6SC04634G-s001.pdf]

Electronic Supplementary Material (ESI) for Chemical Science.
This journal is © The Royal Society of Chemistry 2016

Supporting Information

Covalent tethering photo-responsive superficial layers on hydrogel surfaces for photo-controlled release

Lie Chen[a], Xi Yao[b], Zhandong Gu[a], Kaikai Zheng[c], Chuangqi Zhao[a], Wenwei Lei[a], Qinfeng Rong[a], Ling Lin[g], Jiaobing Wang[d], Lei Jiang[ae] and Mingjie Liu[af]*

[a] *Key Laboratory of Bio-Inspired Smart Interfacial Science and Technology of Ministry of Education, School of Chemistry and Environment, Beihang University, Beijing, 100191, P. R. China.*
[b] *School of Engineering and Applied Sciences, Kavli Institute for Nanobio Science and Technology, Harvard University, Cambridge, 02138 MA, USA.*
[c] *State key laboratory of Polymer Physics and Chemistry, Institute of Chemistry, Chinese Academy of Sciences, Beijing, 100190, P. R. China.*
[d] *School of Chemical and Chemistry Engineering, Sun Yat-sen University, Guangzhou, 510275, P. R. China.*
[e] *Key Laboratory of Bio-inspired Materials and Interfacial Science, Technical Institute of Physics and Chemistry, Chinese Academy of Sciences, Beijing 100190, P. R. China.*
[f] *International Research Institute for Multidisciplinary Science, Beihang University, Beijing, 100191, P. R. China*
[g] *Engineering Research Center of Marine Biological Resource Comprehensive Utilization, SOA, The Third Institute of Oceanography of the State Oceanic Administration, Xiamen 361005, China*

1. Materials and general methods:

All of the following chemical reagents were used as received from Sigma-Aldrich [dimethyl amino proyl acrylamide (DMAPAAm), dimethyl acrylamide (DMAAm), potassium persulfate (KPS), N, N, N', N'-tetramethyl ethylene diamine (TEMED)], clay nanosheets (Laponite XLS, Rockwood), Sinopharm Chemical Reagents [dichloromethane (CH_2Cl_2), 1,2-dichloroethane, ethanol, acetone, sodium hydroxide], Fluorescein was used as received from TCI, deionized water was obtained via Milli-Q. All photo images in the current work were taken by the photo camera (Cannon 60D). Planetary centrifugal Mixer (THIKY Mixer, ARE-310) was used to obtain the transparent and homogeneous precursor of hydrogel. All contact angles on sample surfaces were measured using the contact angle measuring system (OCA20, Dataphysics). The thickness of modified layer was characterized by laser scanning confocal microscope (LSM 780, Zeiss). The surface topography of hydrogel was characterized by 3D optical microscopy (Contour GT-K, Bruker). The FTIR was characterized by infrared spectrum (VERTEX 80, Bruker). The fluorescence spectrum was characterized by Fluorescence Spectrophotometer (Cary Eclipse, Varian). The substance diffusion process was monitored and recorded by UV spectrometry (UV-3600, Shimadzu). The light intensities of the UV light-emitting diode (LED) (OMRON ZUV-C30H, 365 nm) and the vis-LED (CCS HLV-24, 470 nm) for irradiation were 120 mW cm^{-2} and 30 mW cm^{-2}, respectively.

2. Preparation of hydrogel

Hydrogel was polymerized by using KPS as initiator and clay nanosheets as crosslinker. Generally, DMAPAAm and DMAA (anchoring monomer and regular monomers, respectively, with the ratio of 10:1) was first dissolved in water (for the purpose of protecting the tertiary amine groups on anchor monomers, NaOH was used to obtain a alkaline solution pH ~ 11), The total amount of monomers was 20 wt% (to water) in the experiment. Then clay nanosheets (Laponite XLS) (5 wt% ~15 wt% of water amount) was added and the whole mixture was mixed

with Planetary centrifugal Mixer until fully transparent solution was obtained. The solutions were placed in an water bath, at about 5 ℃ for 10 minutes (to prevent the occurrence of implosion), The polymerization were initiated by KPS (0.005 mol/L) and catalytic by TEMED (0.013 mol/L). The resultant hydrogels were rinsed by ample water to remove residual monomers. For further experiment usage, the as-prepared hydrogel was chopped into hydrogel dices (1 by 1 by 0.5 cm^3).

3. Post-modulation on hydrogel surfaces

A hydrogel cube was immersed in the dichloromethane, in which a certain concentration of modifiers was dissolved (0.5 mg/mL). To prevent decomposition of IBSP, the quaternization reaction was carried out in the dark environment. The hydrogel dices were immersed for a certain time about 6 ~ 12 hours, which turn out was the optimized condition for the surface modification. For further experiment usage, the as-prepared hydrogels were rinsed with excessive CH_2Cl_2 in order to remove physically attached modifiers, then dried by nitrogen flow. The as prepared gel pieces were stored at low temperature (2 ~ 8℃, to slow down the dehydration of hydrogel) in the dark.

4. Contact angle measurements

Contact angles (CAs) were measured on an OCA20 system (Dataphysics, Germany) at room temperature (25 ℃), for water contact angle measurement, 2 μL droplet of water was syringed out and dropped onto the underlying surfaces in air. For the underwater oil (1,2-Dichloroethane) contact angle measurement, 2 μL droplet of 1,2-Dichloroethane (DEC) was syringed out and dropped onto the surface of hydrogel in aqueous environment. The average CA values were obtained by measuring at least five different positions on the same sample.

5. Adhesion force measurements

The adhesion force was measured by using a high-sensitivity microelectromechanical balance system (Dataphysics DCAT 11, Germany). The force was measured in aqueous environment. A DEC droplet about 10μL was suspended with a copper cap first, and a hydrogel sample (1 by 1 by 0.5 cm^3) was placed on the balance table, the substrate moved upward at a constant speed of 0.005 mm s^{-1}, until the underlying surface contacted the DEC droplet. Then the surface was moved down. Adhesion forces were obtained from the force-distance curves. For each sample, the average adhesion force was obtained from five repeats.

6. Preparation of fluorescein-incorporated hydrogel

In order to measure the diffusion rate of substance from hydrogel to the water environment, we used fluorescein as the diffusing substance, whose concentration in water can be detected and monitored by UV-Vis spectroscopy. In the experiment, 5 mg of fluorescein was dissolved in 100 mL of water. For preparing hydrogel, $K_2S_2O_8$ and TEMED were utilized as the initiator and catalyst, respectively. In the aqueous solution, molar ratio between monomers, initiator and catalyst (TEMED) was kept at 100 : 0.426 : 0.735. Because the polymerization rate increases as increasing temperature, the solution containing monomer, clay and fluorescein was kept at ca. 5˚C before adding initiator and catalyst. After adding initiator and catalyst, the solution was kept at room temperature for 24 h. The resultant hydrogels were rinsed by ample water to remove residual monomers.

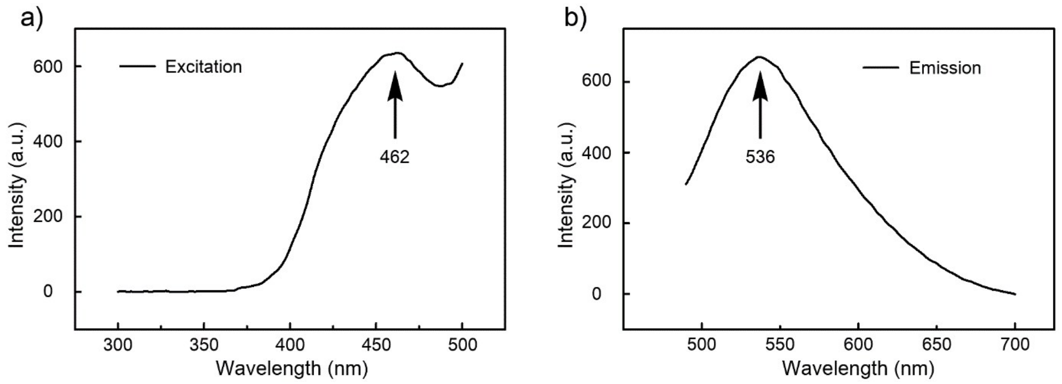

Figure S1. Fluorescent property of the IBSP molecules. a), fluorescence excitation spectrum. b), fluorescence emission spectrum.

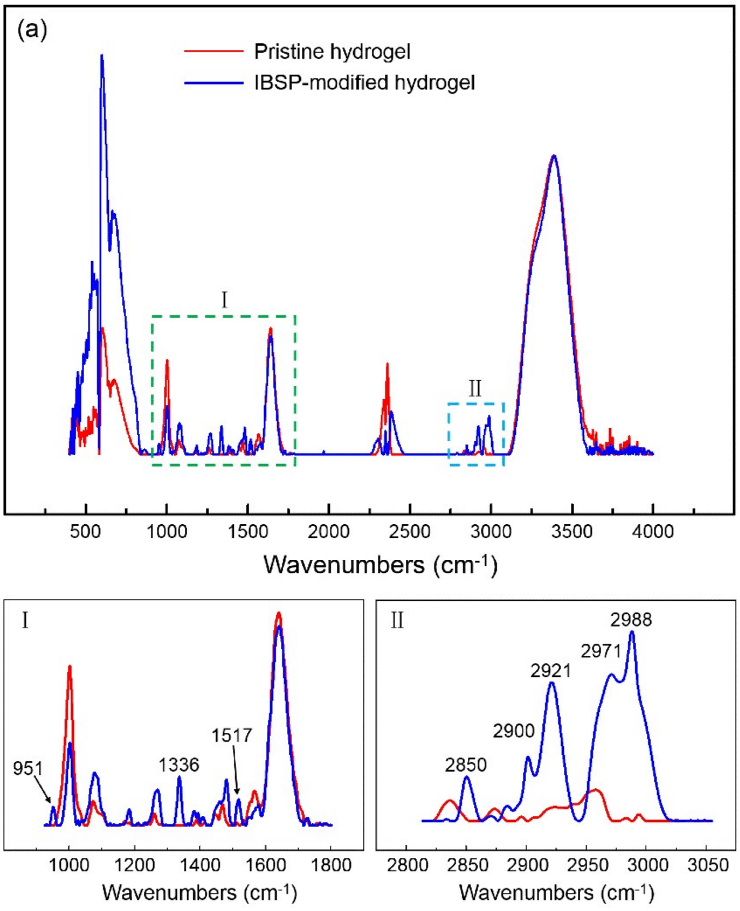

Figure S2. (a) FTIR data of hydrogel surfaces before and after surface modification. (I) *Finger print region*, the peak at 951 cm^{-1} represent the O-C-N bond in spiropyran, the appearance of peaks at 1336 cm^{-1} and 1517 cm^{-1} can be attributed to the nitro group (NO_2) of IBSP structure [1]. (II) *C-H region*, the vibration absorption peaks near to 2900 cm^{-1} can be ascribed to C-H stretching vibration [2].

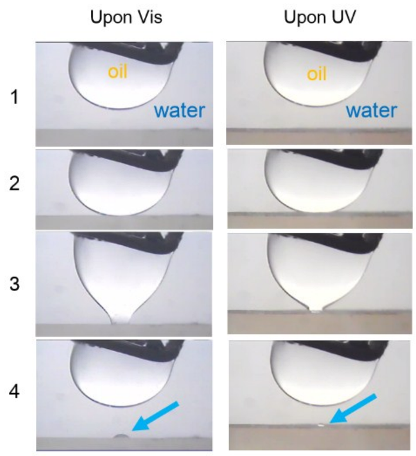

Figure S3. Photographs of four processes during underwater dynamic oil-adhesion test before (left) and after (right) UV irradiation. Process 1: hydrogel surface approaches the oil droplet; process 2: hydrogel surface contact with oil droplet; process 3: hydrogel surface leaves the oil droplet; process 4: hydrogel surface breaks away from the oil droplet. Upon visible light illumination the stretching degree of oil droplet is more severe (process 3), and the amount of residual left on the hydrogel surface is larger (process 4), indicates a larger adhesion force compare with illuminated by UV light.

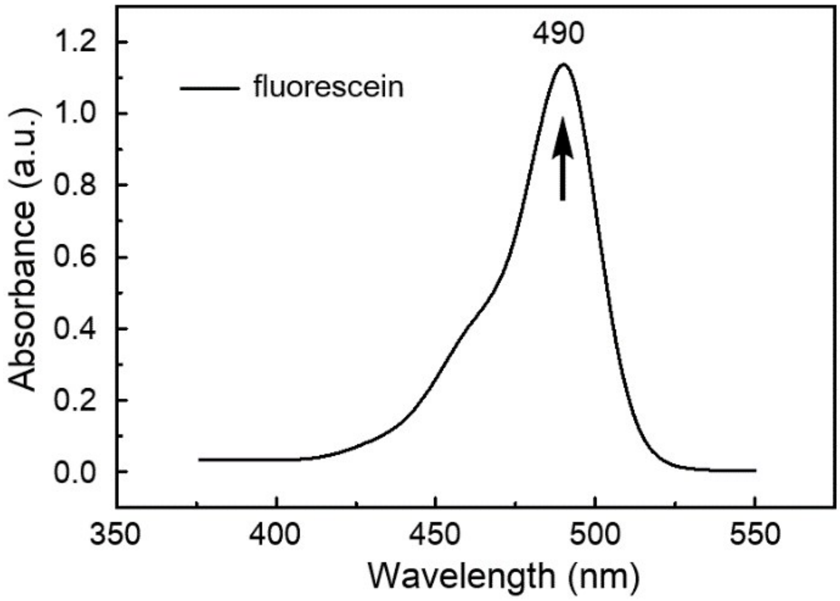

Figure S4. The UV-Vis absorption spectrum of fluorescein.

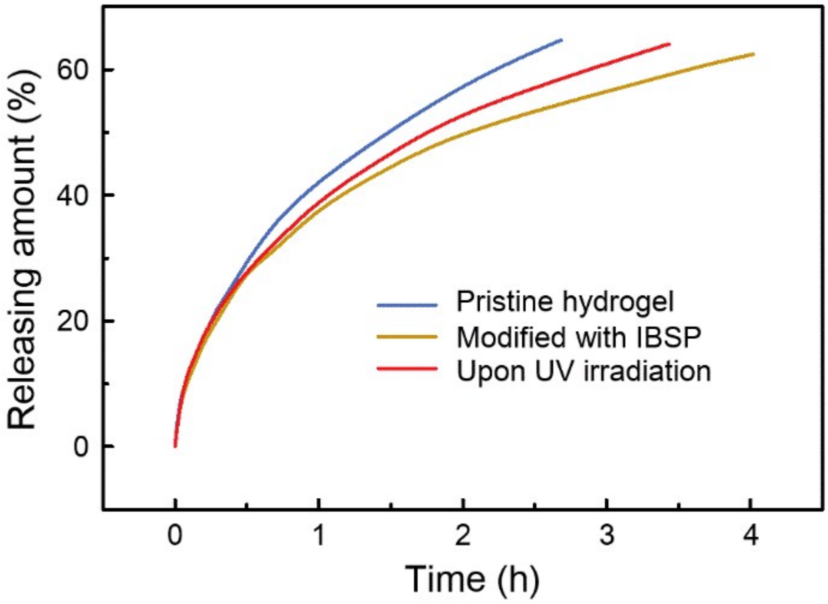

Figure S5. The in situ UV absorption at 490 nm as a function of time, shows the fluorescein released to the water environment from the hydrogels from beginning to about 60% of the total fluorescein amount released from hydrogel.

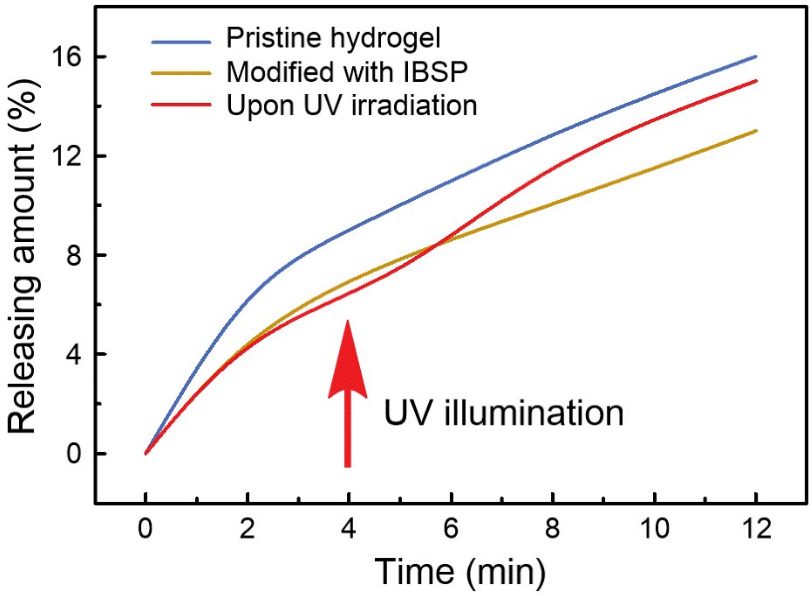

Figure S6. The first 12 minutes of fluorescein release of Figure S5. The UV illumination was applied after 4 minutes from the beginning. The release of fluorescein from the IBSP-hydrogel was accelerated after UV irridiation.

REFERENCES

[1] X. Tian, B. Wang, J. Li, J. Zeng, K. Chen, BioResources, 2016, 11(4), 8627-8637.

[2] R. Delgado Macuil, M. Rojas Lopez, M. Bibbins Martinez, V. Camacho Pemas, AIP CONFERENCE PROCEEDINGS, 2008, 992, 1237-1241.
